# Difenoconazole Degradation by Novel Microbial Consortium TA01: Metabolic Pathway and Microbial Community Analysis

**DOI:** 10.3390/ijms26073142

**Published:** 2025-03-28

**Authors:** Tianyue Wang, Jingyi Sui, Yi Zhou, Liping Wang, Jia Yang, Feiyu Chen, Xiuming Cui, Ye Yang, Wenping Zhang

**Affiliations:** Key Laboratory of Sustainable Utilization of Panax Notoginseng Resources of Yunnan Province, Faculty of Life Science and Technology, Kunming University of Science and Technology, Kunming 650500, China; wangtianyue26@163.com (T.W.); suijingyi29@163.com (J.S.); zhouyi001016@163.com (Y.Z.); lpwang0301@126.com (L.W.); jia091502@163.com (J.Y.); chenfeiyv2002@163.com (F.C.); cuixm@kust.edu.cn (X.C.)

**Keywords:** difenoconazole, biodegradation, microbial consortium, degradation pathway, bioremediation

## Abstract

Difenoconazole, a broad-spectrum systemic fungicide, can effectively prevent and control plant diseases such as rice blast, leaf spot, and black spot caused by *Colletotrichum godetiae*, *Alternaria alternata*, and *Neopestalotiopsis rosae*. However, its residual accumulation in the environment may pose potential toxicity risks to non-target organisms. In this study, a highly efficient DIF-degrading microbial consortium TA01 was enriched from long-term pesticide-contaminated soil by a laboratory-based adaptive evolution strategy. The microbial consortium TA01 was able to degrade 83.87% of 50 mg/L of DIF within 3 days. In addition, three intermediate metabolites were identified using HPLC–MS/MS, and the results indicated that the degradation of DIF by microbial consortium TA01 may involve catalytic reactions such as hydrolysis, dehalogenation, and hydroxylation. High-throughput sequencing results showed that *Pantoea*, *Serratia*, *Ochrobactrum*, and *Bacillus* were the dominant microbial members involved in the degradation process. Finally, bioremediation capacity experiments showed that inoculation with microbial consortium TA01 was able to accelerate the degradation of DIF in the water–sediment system. The findings of this study not only enrich the microbial resources available for DIF degradation but also offer new potential strategies for in situ remediation of DIF contamination.

## 1. Introduction

Difenoconazole is a broad-spectrum triazole fungicide which can effectively prevent and control a variety of plant diseases by inhibiting fungal mycelial growth and conidium formation. Its targets cover the major pathogen groups, such as Ascomycetes, Ascomycetes, and Hemipterans, and are as follows: strong killing activity against sensitive strains of *Colletotrichum godetiae* [1]; significant inhibition of black spot disease of ootheca [2] and black stem disease of quinoa [3] caused by *Alternaria caulina* [1]; significant inhibition of black spot disease of auburn caused by *Alternaria alternata* [2] and black stem disease of quinoa caused by *Alternaria caulina* [3]; and significant prevention of root rot of strawberry caused by *Neopestalotiopsis rosae*, which could recover up to 70% of the yield loss [4]. The agent also controls pea powdery mildew (*Erysiphe pisi*) by interfering with conidiogenesis [5], and controls apple blotch (*Diplocarpon coronariae*) by inhibiting mycelial expansion [6] and potato brown leaf spot (*Phaeosphaeriopsis obtusispora*) [7]. Its broad spectrum is further reflected in the significant inhibition of cross-species pathogens such as *Fusarium* spp., *Didymella segeticola*, and *Mucor xinjiangensis* [8,9,10]. Field data have shown that this agent has a stable effect in the control of major diseases such as rice blast (*Magnaporthe oryzae*) and maize leaf spot (*Magnaporthiopsis maydis*) [11,12], which emphasizes its key role in the integrated management system of crop diseases [11].

In contrast, the environmental residues of DIF pose a multidimensional risk to non-target organisms and ecosystems. In terms of the mechanisms of toxicity, the compound can cause cross-species toxicity by inducing oxidative stress, interfering with metabolic pathways and activating apoptotic signaling. For example, in zebrafish, it has caused motor dysfunction by inhibiting neurotransmitter levels, key enzyme activities, and neurodevelopmental gene expression [13]; in carp, it has caused a significant increase in intestinal oxidative stress and apoptosis [14], as well as triggering organ damage such as glomerular edema and gill tissue necrosis [15]; and in mammals it manifests as disruption of endocrine function—inhibition of human 3β-HSD1 enzyme activity leads to reduced luteinizing hormone synthesis [16]—and induces ER stress and inflammatory responses in bovine mammary epithelial cells [17]. At the ecological level, DIF has significantly altered the structure of the gut flora of soil organisms (e.g., *Enchytraeus crypticus*), reduced the abundance of beneficial bacteria [18], and inhibited the functioning of the honeybee gut microbiome [19], disrupting the microbial ecological balance of soil organisms. In terms of environmental residues, it has been detected in the surface water of the Yangtze River Delta estuary in China at a concentration of 0.412 ± 0.424 μg/L [20], and has shown a trend towards enrichment in nearshore sediments [21]. In terms of agricultural safety, this agent not only inhibits photosynthesis and interferes with glutathione metabolism, hormone signaling, and other key pathways in wheat [22], but also reduces soluble sugar content, increases acidity, and induces oxidative damage in strawberries, which can significantly deteriorate the flavor quality of the fruit [23]. The above evidence suggests that the environmental behavior of DIF has created a “biotoxicity-ecological imbalance-food safety” chain of risks, and that there is an urgent need to weigh the benefits of control against the potential hazards in application management. 

To address the problem of persistent residues of DIF in the environment, a variety of degradation technologies with potential applications have been developed. Among the physicochemical methods based on the adsorption principle, biochar showed 65.3% adsorption of DIF [24], while chitosan biopolymer also demonstrated significant pesticide adsorption capacity through surface functional group complexation [25]. In the field of bioremediation, although the earthworm-mediated bioavailability process can achieve a degradation rate of 42.0% [24], there is still room for improvement in its efficiency. It is noteworthy that microbial degradation technology has become the focus of research because of its high efficiency and environmental friendliness. *Pseudomonas putida* A-3 strain degraded DIF up to 75.98% under optimized conditions [26] and even more groundbreaking is the synergistic bacterial flora system constructed by *Klebsiella*, *Pseudomonas* and *Citrobacter*, which not only promotes plant growth and development but also achieves 85.83–96.59% degradation efficiency of 50 mg/kg of contaminated soil within 45 days in the soil–plant system [27]. These studies indicate that a joint remediation strategy based on microbial community function regulation, which realizes the efficient degradation of pesticides and ecological restoration function at the same time, may become an important direction for future environmental management.

In this study, the construction and mechanisms of microbial degradation systems were investigated systematically in response to the lack of information regarding microbial degradation mechanisms and resources for DIF. An efficient DIF-degrading bacterial population was firstly enriched and screened by a multi-gradient pressure domestication method, and a single factor was used in order to obtain the optimal degradation conditions. On this basis, 16S rRNA high-throughput sequencing was applied to analyze the dynamic succession of microbial consortium structure during the degradation process. The degradation intermediates were traced by high performance liquid chromatography–tandem mass spectrometry (HPLC-MS/MS) to elucidate the metabolic pathway of DIF degradation by bacteria. Finally, the actual remediation efficacy of the microbial consortium on DIF contamination in a composite water–sediment system was assessed by bioremediation simulation experiments. This study provides a theoretical basis and practical foundation for revealing the law of DIF degradation by microbial consortia and developing green bioremediation technology.

## 2. Results and Discussion

### 2.1. Determination of the Ability of Microbial Consortium to Degrade DIF

**Figure 1:**
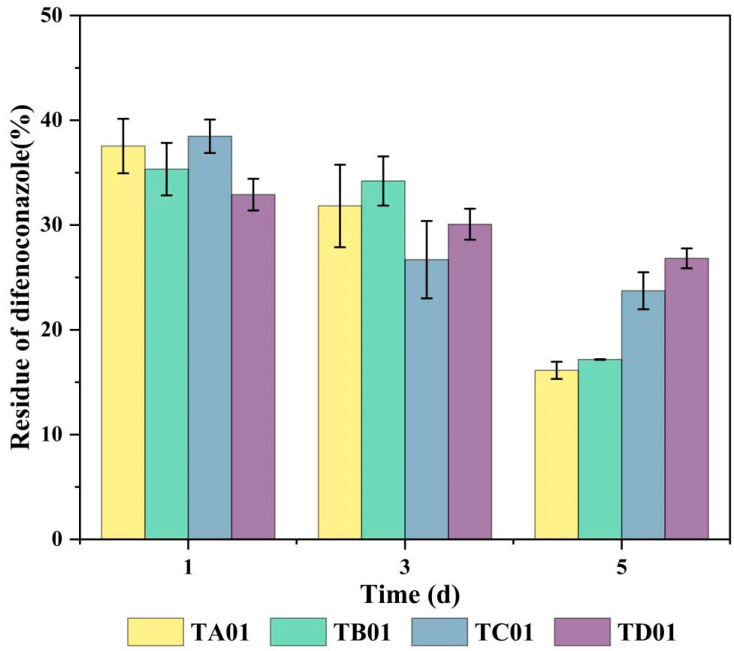
Degradation effect of different microbial consortia on difenoconazole. The bar represents the means of three identical replicates (*N* = 3) and the error line represents the standard deviation from the mean.

In this study, four microbial consortia—TA01, TB01, TC01, and TD01—were screened from four DIF-contaminated soils by enrichment culture. Dynamic monitoring showed (Figure 1) that all four microbial consortia showed some DIF degradation ability in MSM media with 50 mg/L of DIF, with the highest degradation efficiency belonging to microbial consortium TA01 and a relatively slow degradation rate shown by microbial consortium TD01. In addition, the degradation rate for microbial consortium TA01 was 83.87% after 5 days of incubation, which was significantly higher than that for the other microbial consortia (Consortium B: 82.84%, Consortium C: 76.27%, and Consortium D: 73.18%); therefore, microbial consortium TA01 was established as the core flora resource for the subsequent study.

Compared with the reported degradation rate of 62.57% by *Pseudomonas putida* A-3 on 50 mg/L of DIF in 7 d under optimal conditions [27], the present study’s microbial consortium TA01 was able to achieve 83.87% degradation in 5 d at the same concentration. In addition, two strains of bacteria—strain T-1 (*Phyllobacterium* sp.) and strain T-2 (*Aeromonas* sp.)—were reported to be able to achieve 96.32% when cultured for fourteen consecutive days (concentration of 20 mg/L) [28]. Although the final degradation rate of these two strains appeared to be higher, when analyzed in the time dimension, microbial consortium TA01 (mainly composed of *Pantoea*, *Serratia*, *Ochrobactrum*, and *Bacillus*) was able to reach a higher degradation rate in a shorter period of time, reflecting a more efficient degradation ability. It can be seen that a variety of microorganisms within each microbial consortium can cooperate with each other and act on DIF from different pathways at the same time, thus accelerating the degradation process and improving degradation efficiency; whereas, due to the single metabolic pathway of a single bacterium, the degradation rate tends to be slower and the degradation effect is not significant.

### 2.2. Curves of Growth and Degradation Relationship of Microbial Consortium TA01

**Figure 2:**
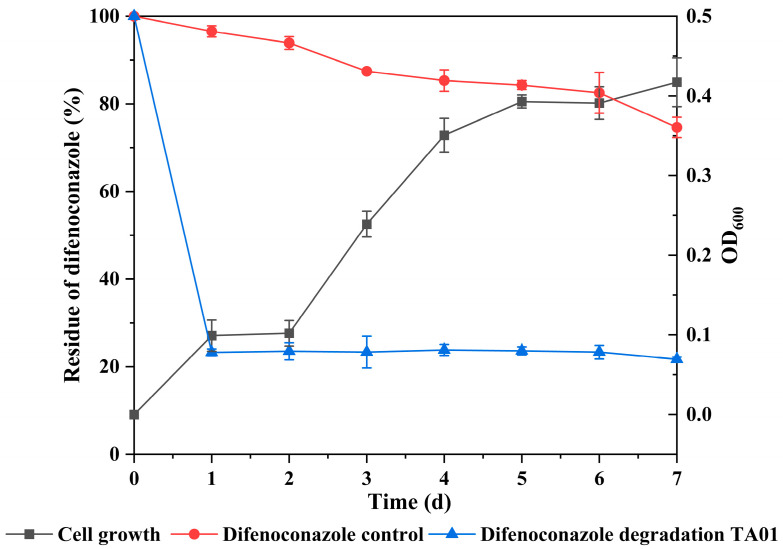
Degradation of difenoconazole during the growth of microbial consortium TA01. Dots indicate the means of three identical replicate samples (*N* = 3) and error lines indicate the standard deviation from the mean.

In view of the fact that microbial consortium TA01 has a superior degradation ability to the other three groups, this study further investigated the growth and degradation relationship. In order to investigate the relationship between growth and degradation for microbial consortia during the degradation of DIF, the growth and degradation rate of microbial consortium TA01 during the degradation of DIF were also monitored. The results, as shown in Figure 2, showed that microbial consortium TA01 was able to rapidly degrade DIF and was able to utilize DIF as the only source of carbon to participate in the metabolic process of the microbial consortium, which could be converted into the nutrients and energy materials required for its growth and metabolism. As can be seen from Figure 2, the microbial consortium TA01 did not have an obvious delay period and achieved rapid degradation of DIF on the first day, with a degradation rate of 76.73%. The DIF degradation rate did not vary much with the continuous growth of microbial consortia and reached 78.31% on day 7. However, there was only weak degradation of DIF in the control group (25.37%).

For the phenomenon that occurs in Figure 2, this study suggests the following possible causes: At the beginning, the concentration of DIF was sufficient, and the microbial consortium rapidly utilized it as a carbon source for degradation; however, the substrate residue decreased significantly after 2 days, and the degradation reaction was limited by the amount of substrate available, which led to the slowing down of the degradation rate, although the change in degradation rate was not obvious. At the same time, after an acclimatization period, the microbial consortium tends to mature in terms of reproduction conditions (e.g., nutrient transformation, metabolic environment) and enters into a logarithmic growth period, showing exponential growth. On the other hand, the microbial consortium may have activated other metabolic pathways, such as utilization of intracellular storage material and synergistic utilization of other carbon and nitrogen sources in the system, in parallel with the degradation of DIF. These pathways prioritize the growth requirements of the bacterial population so that growth is not limited by the DIF degradation efficiency, leading to an eventual segregation phenomenon of a smooth degradation rate and an exponential growth rate.

### 2.3. Effect of Culture Conditions on the Degradation of DIF by Microbial Consortium TA01

**Figure 3:**
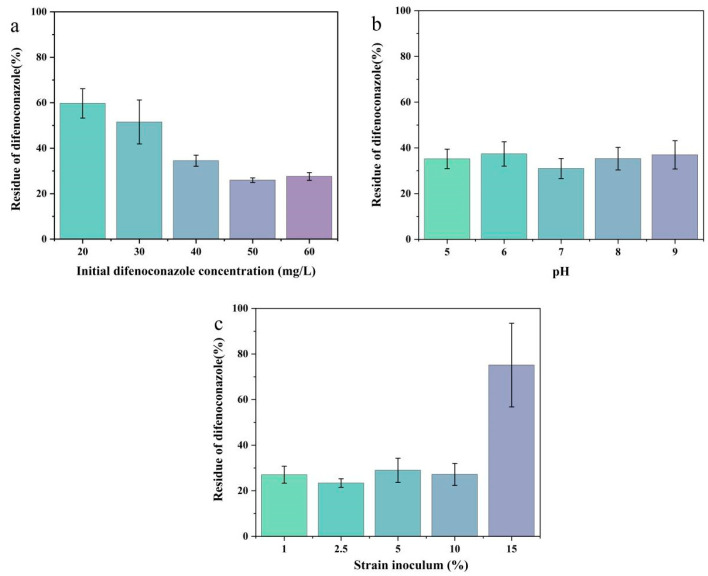
Effect of initial concentration (**a**), pH (**b**), and inoculum (**c**) of difenoconazole on the degradation of difenoconazole. The bar represents the means of three identical replicates (*N* = 3) and the error line represents the standard deviation from the mean.

In this experiment, the effects of pH, inoculum size, and initial concentration of DIF on the degradation of DIF by microbial consortium TA01 were investigated using one-way optimization. The effect of the initial concentration of DIF on the degradation of DIF by microbial consortium TA01 is shown in Figure 3a. When the initial concentration of DIF was 50 mg/L, microbial consortium TA01 had the highest DIF degradation rate, which amounted to 74.06% at 3 d. The degradation rate of microbial consortium TA01 was 74.06%. However, the degradation rate kept decreasing when the concentration of DIF was higher than 50 mg/L.

This may be due to the fact that at low initial concentrations, DIF can induce the expression of biodegradation-related genes to produce a large number of enzymes, thus promoting the metabolism of DIF. However, high concentrations of DIF not only inhibit the growth of the strain but also have a toxic effect on the enzymes involved in microbial degradation, thereby inhibiting enzymatic reactions. This is consistent with the findings of previous studies such as by Tan et al., who found that the degradation rate of chloramphenicol by *Aeromonas media* SZW3 increased gradually in the range of 5–10 mg/L and decreased significantly when above 20 mg/L [29]. In addition, the degradation rate gradually decreased in the range of 20–100 mg/L for strain T-1 (*Phyllobacterium* sp.) and strain T-2 (*Aeromonas* sp.), as studied by Chen et al., who also studied DIF [28]. It can be seen that microbial consortium TA01 (mainly composed of *Pantoea*, *Serratia*, *Ochrobactrum*, and *Bacillus*) in the present study can withstand a higher degradation concentration than the single strain with good degradation.

The degradation of DIF is critically important. The pH affects the activity of microbial enzymes, which in turn affects the growth and metabolic processes of the bacteria. As can be seen in Figure 3b, the DIF degradation efficiency was shown to be high between pH 6 and 7. At pH 7, the degradation rate of microbial consortium TA01 was 69.04%. Too high or too low pH affects the growth and enzyme activity of the bacteria. Many experimental studies have shown that microorganisms generally prefer neutral environments, e.g., strain T-1 (*Phyllobacterium* sp.), strain T-2 (*Aeromonas* sp.) [28], and *Pseudomonas putida* A-3 [26]. This may be due to the fact that a neutral environment is a mild degrading environment where microorganisms can adapt more quickly and thus degrade pesticides better.

The effect of inoculum amount on the degradation of DIF by microbial consortium TA01 is shown in Figure 3c. The DIF degradation efficiency of microbial consortium TA01 increased gradually with the increase in inoculum amount. When the inoculum amount was 2.5%, microbial consortium TA01 showed the highest degradation efficiency for DIF, and the degradation rate reached 76.6% at 3 d. The degradation efficiency of microbial consortium TA01 was higher than that of microbial consortium TA01 when the inoculum amount was 2.5%. When the inoculum amount was increased to 20%, its degradation efficiency did not show significant increase, probably because the growth of the bacteria was too fast and the amount of substrate was not enough to meet the growth and metabolism of the bacteria. When the inoculum is too low, the concentration of bacteria is too low, the growth delay period is prolonged, the cell activity is low, and the time required to reach the appropriate degradation efficiency is longer.

While strain T-1 (*Phyllobacterium* sp.) was reported to achieve its maximum degradation rate at only 5% inoculum (83.25%), strain T-2 (*Aeromonas* sp.) was reported to achieve maximum degradation rate at 10% inoculum (80.28%) [28], which required more biomass than microbial consortium TA01 (mainly composed of *Pantoea*, *Serratia*, *Ochrobactrum*, and *Bacillus*) required to achieve maximum degradation rate at 2.5% inoculum (76.6%). This can be seen in the strong degradation potential of the bacteria at lower biomass inputs. This may be due to the existence of synergistic effects between microorganisms within the microbial consortium, which allows efficient activation of the expression of degradation-related genes and enzymatic activity at low starting numbers, leading to efficient metabolism of pollutants.

### 2.4. DIF Degradation Kinetics Analysis

**Figure 4:**
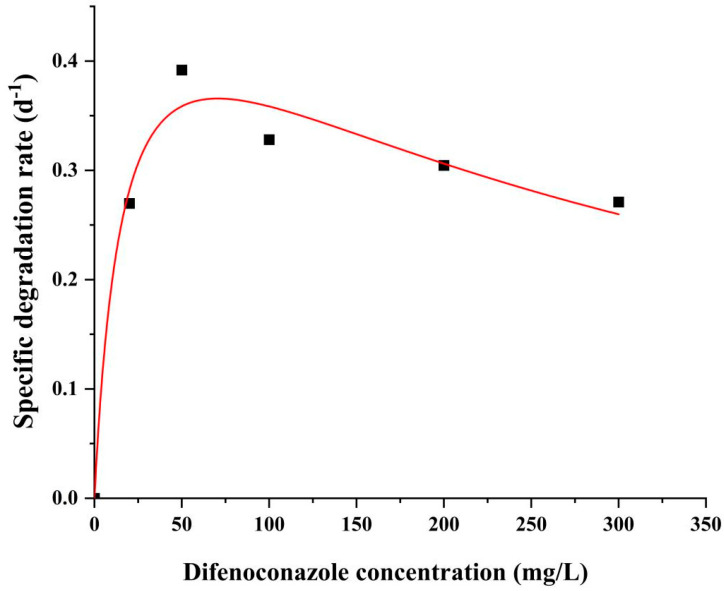
DIF degradation kinetics of TA01 for different initial concentrations: Relationship between the initial difenoconazole concentration and specific degradation rate.

During co-metabolism, primary substrate concentration may appear to inhibit microbial growth and substrate degradation kinetics can be modeled using the Andrews equation [30,31,32]. As shown in Figure 4, in this study, the following kinetic parameters were obtained by nonlinear fitting of the experimental data with Origin 2024: maximum specific degradation rate *q_max_* = 0.5 d^−1^; half-saturation constant *K_s_* = 17.238 mg/L; inhibition constant *K_i_* = 289.344 mg/L; and model coefficient of determination R^2^ = 0.97498. Through the derivation of the equation, the maximum specific degradation rate (*S_max_*) corresponding to the initial concentration of DIF was calculated to be 70.63 mg/L, which was the optimal initial concentration for the degradation of DIF by microbial consortium TA01.

As shown in Figure 4, when the concentration of DIF was lower than 70.63 mg/L, the specific DIF degradation rate of microbial consortium TA01 increased rapidly with the increase in the initial concentration; when the initial concentration was more than 70.63 mg/L, the specific degradation rate decreased with the increase in the initial concentration, which showed the substrate inhibition effect. This may be due to the high concentration of DIF, which produces toxicity to the microbial consortium and inhibits its growth.

### 2.5. Community Structure of Degradative Flora and Analysis of Key Degradative Bacteria

In order to analyze the key genera contributing to DIF degradation by microbial consortium TA01, the present study was carried out to investigate the community changes in the process of DIF degradation by microbial consortium TA01 by utilizing 16S rDNA high-throughput sequencing technology. As can be seen from Table 1, the sequence numbers of all sequenced samples range from 80,451 to 87,525. The α-diversity of microbial consortium TA01 showed a phased change: the Chao1 index decreased from an initial 257.05 to 77.12 and then rebounded to 97.00, and the Shannon index decreased from 4.51 to 2.23 and then recovered gradually to 2.78. This trend suggests that high substrate concentrations in the early stages of degradation (0–5 d) lead to a decrease in community richness (enrichment of specific degrading bacteria), whereas the accumulation of metabolic by-products in the later stages (5–7 d) activates secondary metabolic flora and facilitates micro-ecological reconfiguration. The Simpson dominance index rebounded to 0.76 after dropping from 0.89 to 0.60, corroborating the adaptive shift from broad-spectrum competition to specialized metabolism.

**Figure 5:**
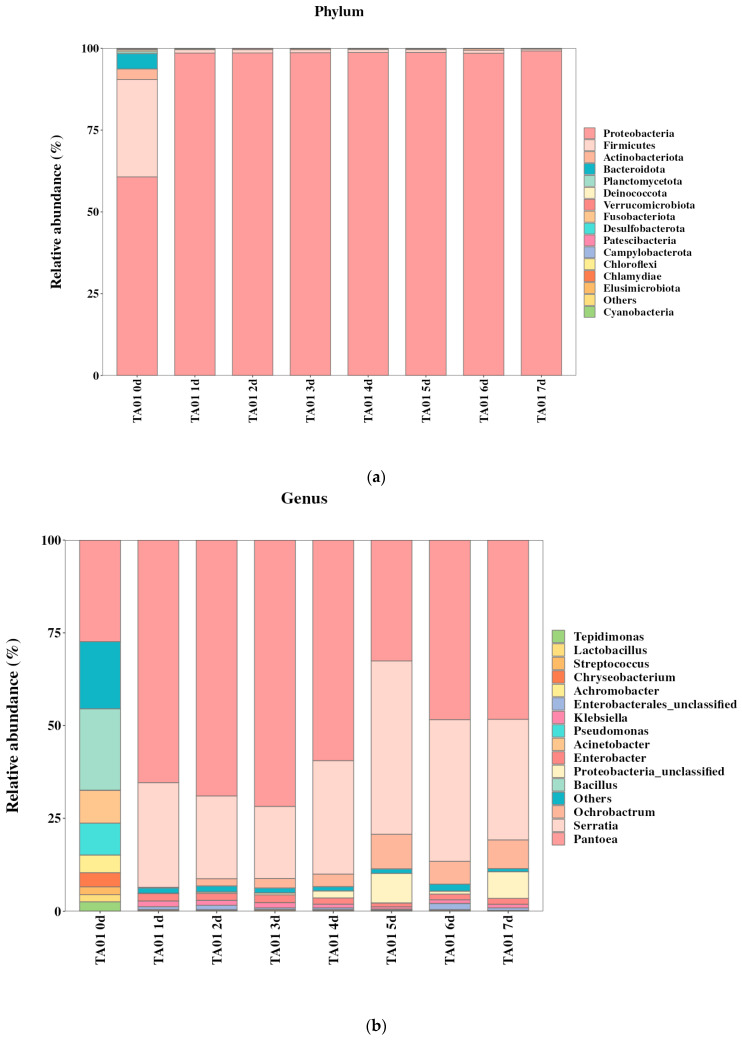
Evolution of microbial consortia at phylum (**a**) and genus (**b**) level during the degradation of difenoconazole.

The results of the phylum and genus level analyses of the community structure during DIF degradation by microbial consortium TA01 are shown in Figure 5. At the phylum level (Figure 5a), in the initial stage, the relative abundance of *Proteobacteria* was 60.71%, which coexisted with *Firmicutes* (29.75%), *Actinobacteriota* (3.23%), and *Bacteroidota* (4.7%). As the degradation process progressed, the abundance of *Proteobacteria* continued to increase to 99.20%, while the abundance of other phyla decreased to <0.5%. The relative abundance of *Proteobacteria* increased significantly during the degradation process, in contrast to a sharp decrease in the relative abundance of *Firmicutes*, *Actinobacteriota*, and *Bacteroidota*. It can be seen that this degradation process of *Proteobacteria* showed a significant metabolic advantage. Researchers have found that species of *Proteobacteria* have important applications in industry, agriculture, medicine, health, and environmental protection, especially in industrial and agricultural wastewater treatment, soil remediation, and pollutant degradation [33].

In addition, genus level analysis (Figure 5b) revealed the dynamics of the degrading genera in microbial consortium TA01: the initial stage was dominated by *Pantoea* (27.38%), *Bacillus* (21.95%), *Pseudomonas* (8.60%), and *Actinobacteria* (8.86%); in the middle stage of degradation, the abundance of *Pantoea* and *Serratia* increased to 71.79% and 19.45%, respectively; and in the end stage, a stable community was formed, dominated by *Pantoea* (48.33%), *Serratia* (32.49%), and *Ochrobactrum* (7.74%). It is noteworthy that the above genera are reported for the first time in the study of DIF degradation, providing candidate resources for the development of novel degrading strains.

### 2.6. Characterization of DIF Degradation Products

In this study, three main DIF degradation products from microbial consortium TA01 were found (Table 2). One of them, DIF-TPs 406, with a mass-to-charge ratio of 406.00 for the hydrogenation peak [M+H] and a molecular formula of C_19_H_20_ClN_3_O_5_, loses one chlorine atom and adds two oxygen atoms compared to the molecular formula of the DIF parent (C_19_H_17_Cl_2_N_3_O_3_), a process that occurs by hydroxylation of halogen atoms and hydrolysis to open the ring [28]. DIF-TPs 330, with a mass-to-charge ratio of 330.25 for the hydrogenation peak [M+H] and a molecular formula of C_16_H_13_ClN_3_O_3_, should be hydrolyzed for the hydrolysis of an oxygen-containing five-membered heterocyclic ring to open the ring and lose a C_2_H_4_O molecule. DIF-TPs 290, with a mass-to-charge ratio of 290.25 for the hydrogenation peak [M+H] and a molecular formula of C_16_H_15_ClO_3_, underwent a hydrolysis reaction and lost an N-containing five-membered heterocycle C_2_H_2_N_3_ [32].

**Figure 6:**
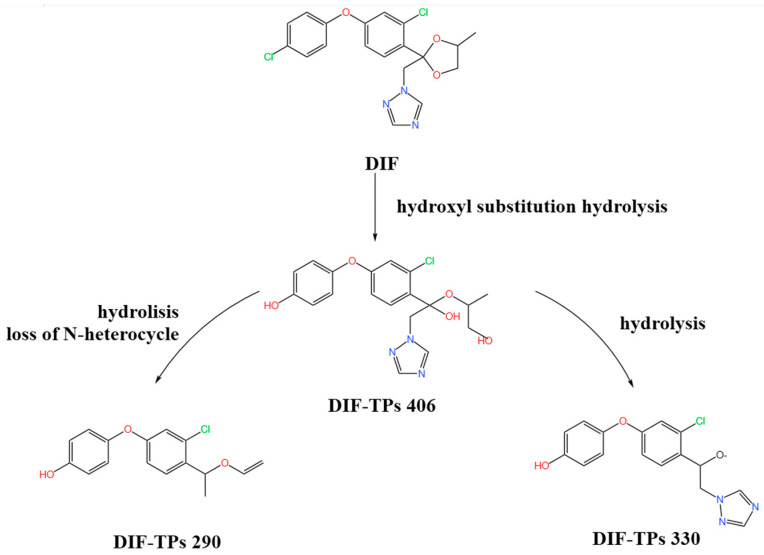
Pathway of difenoconazole degradation by microbial consortium TA01.

Based on the structural identification of the degradation metabolites mentioned above, the DIF biodegradation pathway was deduced in this study, as shown in Figure 6. The parent compound was first generated as the intermediate product DIF-TPs 406 (molecular formula C_19_H_20_ClN_3_O_5_) by hydrolysis and dehalogenation hydroxylation of the dioxycyclohexane structure; subsequently, the oxygen-containing pentaheterocycle of DIF-TPs 406 was further hydrolyzed by ring-opening and the dehydroxylation of the C_2_H_4_O group to form DIF-TPs 330 (C_16_H_13_ClN_3_O_3_); meanwhile, the nitrogen-containing five-membered heterocyclic ring of DIF-TPs 406 was broken and accompanied by a hydrolysis reaction to generate DIF-TPs 290 (C_16_H_15_ClO_3_). This pathway is highly consistent with the reaction mechanisms proposed by Man et al. [33] and Chen et al. [28], suggesting that the core steps of DIF degradation (e.g., ether bond breaking or heterocyclic ring cleavage) are highly similar in different microbial systems [34].

### 2.7. Biodegradation of DIF in Water–Sediment Simulated Pollution Systems

**Figure 7:**
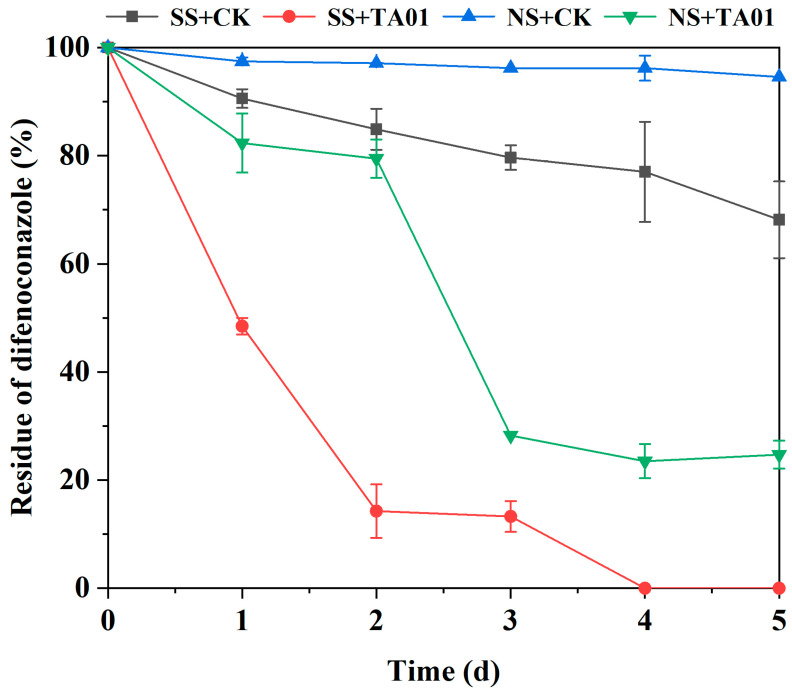
Effect of microbial consortium TA01 on the degradation of difenoconazole in water–sediment pairs.

In order to verify the remediation ability of microbial consortium TA01 on the mixed water–sediment system with DIF contamination, four treatment groups were set up in this section to investigate the bioremediation ability of microbial consortium TA01. The results are shown in Figure 7, from which it can be seen that inoculation of microbial consortium TA01 in both sterilized and unsterilized water–sediment systems significantly increased the DIF degradation rate. The DIF degradation rate by microbial consortium TA01 was 75.29% in the 5 d unsterilized water–sediment system, whereas microbial consortium TA01 reached 100% degradation in the sterilized water–sediment system. This indicates that the microbial consortium TA01 is able to exert its degradation ability by growing and multiplying rapidly in the water–sediment system. In contrast, the degradation of phenoxymetrazole in sterilized and unsterilized water–sediment was 68.16% and 94.56% in the treatment group not inoculated with the microbial consortium, respectively. This may be due to the possible presence of some substances with degradation capacity in the water–sediment system.

In addition, in order to better characterize the remediation ability of microbial consortium TA01 on the water–sediment system, this study utilized a first-order kinetic model to fit the data on the degradation of DIF in water–sediment by microbial consortium TA01, and the results are shown in Table 3. The coefficients of determination R^2^ for the four treatment groups were 0.97974, 0.98891, 0.88518, and 0.86704, respectively. The degradation rate constants (*k*) were 0.07067, 0.81563, 0.03094, and 0.30261 d^−1^, respectively. the degradation half-lives (*t*_1/2_) were 9.8, 0.9, 22.4, and 2.3 d.

## 3. Materials and Methods

### 3.1. Enrichment and Screening of DIF-Degrading Microbial Consortia

Four different soil samples were retrieved from a Chinese herbal medicine plantation area in Kunming, Yunnan Province, and labeled A, B, C, and D. These four soil samples were enriched to create microbial consortia. Enrichment of DIF-degrading microbial consortia was carried out by an incubation procedure described by Bhatt et al. [35]. About 5 g of soil samples were added to an MSM medium containing 50 mg/L of DIF and incubated for 3 d at 30 °C and 200 rpm in a shaker. Meanwhile, 1 mL of the enriched culture was transferred to a fresh MSM medium containing 100 mg/L of DIF after 3 d. Enrichment cultures were serially transferred to fresh enrichment media containing 200, 300, 400, 500, or 600 mg/L of DIF for another 3 d. Four microbial consortia named TA01, TB01, TC01 and TD01 were finally obtained from the soil samples. Among them, microbial consortium TA01 (mainly composed of *Pantoea*, *Serratia*, *Ochrobactrum*, and *Bacillus*) was the one with the highest DIF degradation capacity among the four microbial consortia and, therefore, was selected for subsequent studies on DIF bioremediation.

### 3.2. Determination of DIF Degradation and Growth for Microbial Consortium TA01

The conserved microbial consortium TA01 was inoculated into an LB medium (30 °C, 200 rpm) for overnight activation, and the bacterial fluid was centrifuged at 3800 rpm for 3 min to collect the bacterial body, the supernatant was discarded and resuspended in sterile water and vortexed for 30–60 s. The washing–centrifugation step was repeated 2 times to remove the medium residue. The seed suspension was prepared by resuspending the final bacterial precipitate in sterile water, transferring it to an inorganic salt medium (MSM) containing 50 mg/L of inoculum with 10% DIF, incubating it at 30 °C and 200 rpm for 7 days under light protection (three replicates were set up for each group). Daily samples were taken at regular intervals on days 0–7 of incubation and were used to determine the biomass of the organisms (a fresh MSM was used as a blank control) and the concentration of DIF (an MSM of uninoculated colonies was used as a blank control), respectively. One milliliter of bacterial biomass was characterized by a UV-2600 ultraviolet spectrophotometer (Shimadzu, Kyoto, Japan) to determine the absorbance at 600 nm (OD_600_), and the determination of the concentration of DIF was implemented by referring to the method of Zhou et al. [26].

### 3.3. Effect of Incubation Conditions on the Degradation of DIF by TA01

In this experiment, the effect of microbial consortium TA01 on the degradation of difenoconazole by microbial consortium TA01 was investigated in terms of three factors: initial pH (5.0, 6.0, 7.0, 8.0, and 9.0), inoculum (1%, 2.5%, 5%, 10%, and 15%), and the initial concentration of difenoconazole (30, 40, 50, 60, and 70 mg/L). The absence of inoculum was used as a control with three replicates for each factor. Each factor was incubated at 30 °C and 200 rpm. Samples were taken after 3 d of incubation to detect the residual DIF concentration by HPLC.

### 3.4. Kinetics of DIF Degradation

In order to characterize the kinetic parameters during the degradation of DIF by microbial consortium TA01, the dynamic parameters of the degradation process by microbial consortium TA01 under different substrate concentration conditions were nonlinearly fitted. In this experiment, five concentration gradients of 20 mg/L, 50 mg/L, 100 mg/L, 200 mg/L, and 300 mg/L were set as the only carbon sources. The microbial consortium TA01 was inoculated into the inorganic salt medium (MSM) at 10% inoculum, three replicates were set up for each group, and the uninoculated system was used as a blank control and incubated at 30 °C and 200 rpm for 5 days while protected from light. Based on the results, the DIF degradation kinetics of microbial consortium TA01 at different initial concentrations of DIF was calculated using the Andrews equation. Subsequently, the experimental data were processed by Origin 2024 software to calculate the optimal initial concentration of microbial consortia for degradation of DIF by nonlinear least squares curve fitting of the experimental values according to the Andrews equation. The equations are as follows:

(1) $$q=\frac{q_{max}S}{S+K_{s}+(S^{2}/K_{i})}$$

In its formula, *S* denotes the concentration of DIF (mg/L), *q* denotes the specific degradation rate (d^−1^), *q_max_* denotes the maximum specific degradation rate (d^−1^), *K_s_* denotes the half-rate constant (mg/L), and *K_i_* denotes the inhibition coefficient (mg/L).

### 3.5. Analysis of the Community Structure and Key Degrading Bacteria of Microbial Consortium TA01

The microbial consortium TA01 was inoculated into liquid LB medium and incubated at 30 °C and 200 rpm in a constant temperature shaker under light protection for 12 h. The precipitated bacterial bodies were collected by high-speed centrifugation at 3800 rpm for 5 min, washed with sterile water 2–3 times, and then added with an appropriate amount of inorganic salt medium to prepare the bacterial suspension at a certain concentration. Subsequently, the bacterial suspension was inoculated into an inorganic salt medium containing 50 mg/L of DIF, followed by incubation in a constant temperature shaker at 30 °C and 200 rpm protected from light for 7 d. Samples were taken at 1 d intervals. High-throughput sequencing of 16S rRNA was used to detect the structure and diversity of microbial communities in enrichment culture, and these samples were analyzed by high-throughput sequencing in this study by Hangzhou Lianchuan Biotechnology Co. (Hangzhou, China).

### 3.6. Identification of DIF Degradation Products

The activated and resuspended bacterial solution was added to MSM containing 50 mg/L DIF and allowed to incubate for 3 d at 30 °C and 200 rpm, while protected from light, and 1 mL samples were taken at 0 h, 6 h, 12 h, 24 h, 36 h, 48 h, and 72 h. MSMs not inoculated with bacterial fluids were used as controls. The DIF-degrading metabolites of microbial consortium TA01 were subsequently identified by HPLC–MS/MS [30]. The detection conditions were as follows: mobile phase—acetonitrile; 0.2% formic acid aqueous solution = 70:30 (*v*:*v*); column temperature—30 °C; flow rate—0.4 mL/min; injection volume—20 μL; scanning mode—positive ion mode using full scan mode and Produce Ion mode to collect the data; full scanning time of 5 min, scanning range of 50~416 *m*/*z*; temperature of ion source—450 °C; capillary voltage—4000 V [31]. 

### 3.7. Bioremediation Potential of Microbial Consortium TA01

Soil taken from the depth of unapplied DIF from the farmland of Kunming University of Science and Technology, Yunnan Province, China, was dried and sieved (2 mm). A sieved soil sample of 5 g was taken into a 250 mL conical flask containing 45 mL of MSM medium and a certain volume of DIF was added to give an initial concentration of 100 mg/L of DIF in the system. Then a 10% bacterial solution (5 mL) was inoculated into this water–sediment remediation system and incubated at 30 °C and 200 rpm for 5 d under light protection, and 1 mL of the sample solution was taken every 1 d for the detection of DIF residues. In this experiment, the unadded bacterial solution was used as a control group and both sterilized and unsterilized treatments were conducted for the water–sediment system, so there were a total of four treatments in this experiment: sterilized system + uninoculated (SS + CK); sterilized system + inoculated (SS + TA01); unsterilized system + uninoculated (NS + CK); and unsterilized system + inoculated (NS + TA01). Three replicates were set up for each treatment.

## 4. Conclusions

In this study, the microbial consortium TA01, which can utilize DIF as its sole carbon source, was obtained through targeted enrichment screening, and the optimal degradation conditions were determined by one-factor determination. Based on high-throughput sequencing, the core composition of microbial consortium TA01 was *Pantoea*, *Serratia*, *Ochrobactrum,* and *Bacillus*, which is the first report of the synergistic degradation system of multiple genera in the degradation of DIF and provides an important candidate resource for the development of novel composite degrading bacterial agents. This synergistic degradation system is the first to be reported in the study of DIF degradation, and provides an important candidate for the development of new composite degraders. In addition, water–sediment simulation experiments confirmed the significant remediation efficacy of microbial consortium TA01 on DIF contamination in complex environments (with a removal rate of 75.29%), which verified its potential for application in practical ecological remediation.

## Figures and Tables

**Table 1:** Bacterial community ordinal number and diversity.

Sample (d)	Sequences	Shannon	Simpson	Chao1
0	87,522	4.51	0.89	257.05
1	85,255	2.23	0.64	87.0
2	85,486	2.31	0.62	87.0
3	87,062	2.17	0.60	94.0
4	82,368	2.42	0.68	86.0
5	84,866	2.77	0.79	77.12
6	83,468	2.84	0.75	111.00
7	80,451	2.78	0.76	97.00

**Table 2:** DIF degradation products.

Compounds	Chemical Structures	Molecular Formula	*m*/*z* [M+H]
DIF-TPs 406	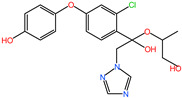	C_19_H_20_ClN_3_O_5_	406.00
DIF-TPs 330	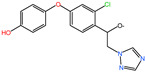	C_16_H_13_ClN_3_O_3_	330.25
DIF-TPs 290	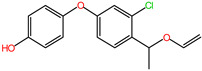	C_16_H_15_ClO_3_	290.25

**Table 3:** Kinetics of DIF degradation by microbial consortium TA01 in water–sediments.

Treatment	Regression Equation	*k* (d^−1^)	*t*_1/2_ (d)	*R* ^2^
SS + CK	C_t_ = 98.77213e^−0.07067t^	0.07067	9.8	0.97974
SS + TA01	C_t_ = 100.84707e^−0.81563t^	0.81563	0.9	0.98891
NS + CK	C_t_ = 99.21675e^−0.03094t^	0.03094	22.4	0.88518
NS + TA01	C_t_ = 106.36416e^−0.30261t^	0.30261	2.3	0.86704

## Data Availability

All data are contained within the article.
